# Can patient and fracture factors predict opioid dependence following upper extremity fractures?: a retrospective review

**DOI:** 10.1186/s13018-019-1233-7

**Published:** 2019-09-26

**Authors:** Vani Janaki Sabesan, Kiran Chatha, Lucas Goss, Claudia Ghisa, Gregory Gilot

**Affiliations:** 1 Levitetz Department of Orthopedic Surgery, Cleveland Clinic Florida, 2950 Cleveland Clinic Blvd, Weston, FL 33132 USA; 2 Charles E Schmidt School of Medicine, Florida Atlantic University, Boca Raton, USA

**Keywords:** Opioid dependence, Proximal humerus fracture, Predictors for opioid dependence, Patient factors

## Abstract

**Background:**

Since the early 1990s, opioids have been used as a mainstay for pain management surrounding fracture injuries. As opioid dependence has become a major public health issue, it is important to understand what factors can leave patients vulnerable. The purpose of this study was to examine what risk factors, patient or injury severity, contribute most to postoperative opioid dependence following surgical treatment of proximal humerus fractures (PHFs).

**Methods:**

A retrospective review of all patients who underwent an open reduction and internal fixation of PHF was performed within a large multisite hospital system. Recorded variables included age, gender, ASA class, BMI, fracture type, time to surgery, pre- and postoperative opioid prescriptions, physical and psychological comorbidities, smoking status, and complications. Pre- and postoperative opioid dependence was defined as prescription opioid use in the 3 months leading up to or following surgery. Odds ratio calculations were performed for each variable, and a multivariate logistic regression was used to compare all predictors.

**Results:**

A total of 198 surgically treated PHFs were included in the cohort with an average age of 59.9 years. Thirty-nine cases were determined to be preoperatively opioid dependent while 159 cases were preoperatively opioid naïve. Preoperative opioid dependence was found to be a significant risk factor for postoperative narcotic dependence, carrying a 2.42 times increased risk. (CI 1.07–5.48, *p* = 0.034). Fracture type was also found to be a risk factor for postoperative dependence, with complex 3- and 4-part fracture patients being 1.93 times more likely to be opioid dependent postoperatively compared to 2 part fractures (CI 1.010–3.764, *p* = 0.049). All other factors were not found to have any significant influence on postoperative opioid dependence.

**Conclusions:**

Our results demonstrate that the most important risk factors of postoperative opioid dependence following proximal humerus fractures are preoperative dependence and fracture complexity. It is important for orthopedic surgeons to ensure that patients who have more complex fractures or are preoperatively opioid dependent receive adequate education on their increased risk and support to wean off of opioids following surgery.

**Level of evidence:**

III

## Background

Currently, approximately 115 Americans die every day from an opioid overdose [1]. Over the last two decades, the number of prescribed opioids, as well as the number of deaths from opioids has increased five-fold [1]. Orthopedic surgeons are the third highest prescribers of opioids in the USA and the highest when compared to other surgical subspecialties [2]. Because orthopedic trauma and related procedures can be extremely painful, patients have often been prescribed excessive opioid-based medications following treatment as no particular guidelines existed until recent legal and regulatory changes have been implemented [3, 4]. Humeral fractures can be very painful and most commonly, pain from this type of fracture and subsequent surgery is managed with opiate-based medications.

It has been well established that preoperative opioid use has been associated with an increased risk of opioid dependence, decreased clinical outcomes and lower patient satisfaction in those undergoing orthopedic surgery [5–14]. Specifically, for orthopedic trauma patients research has found that prolonged postoperative opioid use adversely affects patient-reported outcomes and satisfaction [15, 16]. In addition to preoperative opioid use, other risk factors for increased risk of opioid abuse that have been reported include psychopathology, history of substance abuse, chronic pain, and family history of alcohol and substance abuse [17]. In other fields, screening tools have been developed to aid in recognizing patients that may be at higher risk for opioid abuse, but these are lacking in orthopedic trauma patients [18]. A surgeon’s pre-treatment understanding of risk factors for potential opioid misuse is essential to mitigating the problems associated with these medications.

**Fig. 1:**
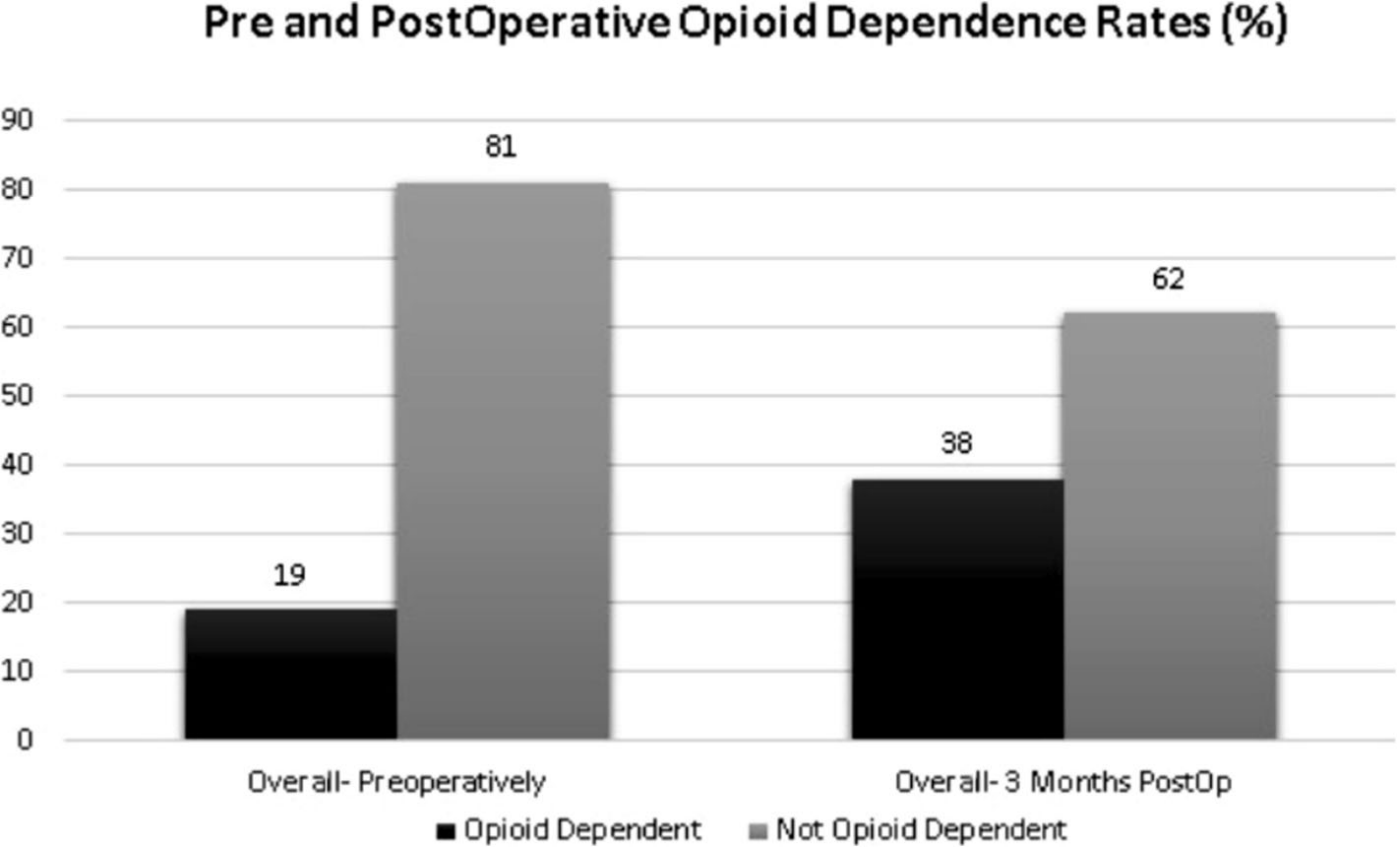
Opioid dependence compared from pre- to post-op

It is unknown whether type of fracture alone is a risk factor, but orthopedic trauma patients have been shown to experience post-traumatic stress syndrome, depression, and anxiety, which are all associated with increased risk for opioid misuse, among other substances [17, 19, 20]. Rosenbloom et al. recently reported 35% rate of opioid usage 4 months after surgery for orthopedic trauma patients which was significantly higher than non-trauma associated postsurgical patients (0.4–3.1%) [4, 21]. Recent literature examining fracture morphology in animal models has also suggested that higher pain is experienced with more bone marrow exposure [22]. The purpose of this study was to examine specific risk factors including patient characteristics and fracture morphology that may influence postoperative opioid dependence in patients undergoing ORIF for proximal humerus fractures. We hypothesized that preoperative opioid consumption would be the strongest predictor of postoperative opioid dependence as this is well established in the literature (Fig. 1).

## Methods

A retrospective review of patients who underwent open reduction internal fixation of proximal humerus fractures (PHF) was performed from 2012 to 2016 in a multisite hospital system. Patients were identified using CPT code 23615 (proximal humerus fracture open reduction and internal fixation (ORIF)). Demographic data collected included age, gender, ASA class, BMI, fracture type, smoking status, psychological, and physical comorbidities. Fractures were classified based on the Neer classification system, and the analysis was performed comparing the 2-part fracture (Simple) group to the 3- and 4-part (complex) fracture group [23]. Patients were categorized based on ASA class (group 1 = class 1 and 2 and group 2 = class 3 and 4) and obesity was defined as a BMI greater than 30. Smoking status was divided based on patients who had a history of smoking and patients who never smoked. Psychological comorbidities were defined as any recorded diagnosis that would be included in the DSM-V [24]. Number of follow-up visits and the amount of time between injury and surgery was also recorded in days.

Opioid prescriptions were recorded for 6 months prior to and 1 year following surgery. All opioid prescriptions were verified using state prescription drug monitoring websites [25, 26]. For each prescription, the medication, dosage, number of pills, and prescribed total morphine equivalents were collected. For patients with multiple opioid prescriptions, an average prescribed total morphine equivalent was calculated. Preoperative and postoperative opioid dependence was defined as continued prescriptions for at least 3 months prior to or following surgery. Total morphine equivalents were compared and analyzed between the pre- and postoperative groups at each time interval.

Descriptive statistics reported the effect of each demographic variable on narcotic dependence. Statistical analyses then compared preoperatively dependent and naïve cohorts using *t* tests and chi-square test for dependence rates and TMEs prescribed. A multivariate logistic regression was performed to identify significant risk factors and calculate postoperative odds ratios for relative risk of dependence associated with each factor. The primary outcome variable of the model was postoperative opioid dependence. A subanalysis of opioid-dependent patients’ prescription TMEs and dependence rates was performed. All statistical analyses were performed using SPSS Software (IBM, Version 23).

## Results

A total of 159 PHFs patients were included in the cohort, with an average age of the 59.9 years and 115 females and 44 males included. Thirty-nine cases were determined to be preoperatively opioid dependent (group 1) while 120 cases were preoperatively opioid naïve (group 2) with no recorded opioid prescriptions in the 6 months leading to surgery. A total of 74 patients were postoperatively dependent on opioids at 3 months. There were no significant differences in age, ASA class, BMI, or psychological comorbidities between the two groups (Table 1). At 30 days postoperatively, 55% of the opioid naïve cohort was able to wean off of opioids while 74% of the preoperatively opioid dependent cohort remained on opioids.

**Table 1:** Comparison of patient demographics in opioid-dependent and naïve groups

Demographics	Opioid dependent *n* = 39	Opioid non-dependent *n* = 120	*p* value
Female gender	28 (72%)	87 (72.5%)	0.931
Psychological comorbidity	20 (51%)	39 (32%)	0.034*
Simple fractures	22 (56%)	72 (60%)	0.691
Mean comparisons between groups			
Age	59.8	59.2	0.292
ASA class	2.64	2.44	0.093
BMI	29.82	27.39	0.14
Procedure time (min)	153.31	156.69	0.953
Time to surgery (days)	19.9	15.47	0.309

* denotes statistically significant differences between groups

Preoperative opioid dependence was found to be a statistically significant risk factor for postoperative opioid dependence, carrying a 2.42 times increased risk. (CI 1.07–5.48, *p* = 0.034). Of the preoperatively naïve group, fracture type was also found to be a risk factor for postoperative dependence, with complex 3- and 4-part fracture patients being 1.93 times more likely to be opioid-dependent postoperatively compared to 2-part fractures (CI 1.01–3.76, *p* = 0.049). There was no influence on postoperative opioid dependence for all other factors in the model including smoking status, ASA class, BMI, or comorbidity burden (Table 2).

**Table 2:** Predictors of postoperative opioid dependence

Predictor	Odds ratio	*p* value
Fracture complexity	1.93	0.049*
ASA class 3 or 4	1.31	0.48
Smoking status	1.38	0.33
Preoperative opioid dependence	2.42	0.03*
Obesity (BMI > 30)	0.60	0.14
Psychological comorbidity	0.93	0.83
Time to surgery > 30 days	0.72	0.46

* denotes statistically significant differences between groups

Those patients that were preoperatively dependent required significantly higher (1.2 times) total morphine equivalents postoperatively (*p* = 0.04). Preoperatively dependent patients received prescriptions from their orthopedic surgeon for a significantly longer period of time, for an average of 80 days following surgery compared to the narcotic naïve cohort who received narcotic prescriptions for an average of 53 days (*p* = 0.03).

## Discussion

Our results demonstrate that the strongest risk factor for postoperative opioid dependence following a proximal humerus fracture is preoperative opioid dependence which is consistent with the orthopedic literature [6, 10, 11, 27]. In addition, patients with preoperative opioid dependence had higher rates of opioid usage after fracture fixation [28, 29]. In orthopedic trauma, studies have found that patients with preoperative opioid use are associated with higher likelihood of prolonged postoperative opioid use and higher rates of doctor shopping [9]. Orthopedic surgeons must educate these patients prior to fracture fixation regarding their increased risks and support better oversite and weaning of these opioid medications postoperatively.

Another risk factor that was identified as an increased risk for opioid dependence was fracture complexity, with more complex fracture types there was nearly a doubled risk of opioid dependence. Pain associated with fractures in the upper extremity has been well documented, and previous literature has found that higher pain levels are associated with poorer clinical outcomes. Bone pain has been studied extensively in all fracture types, and research has shown that increased exposure of the periosteum and marrow cavity has been associated with increased pain sensation [16, 22]. In addition, previous studies have found that higher perceived pain levels are associated with catastrophizing and this may be a contributing factor to higher opioid use [30]. In this study, our results suggest the increased bone marrow exposure of more complex fractures is associated with higher opioid use which may be explained by higher perceived pain.

Surprisingly, there was no association between any demographic variable, except for a slightly higher risk of postoperative opioid dependence for smokers in this population. Considering that a traumatic injury such as a proximal humerus fracture is often the first interaction between an orthopedist and patient, understanding that all patients are susceptible to opioid abuse is important to screen appropriately. Surgeons can feel confident that the majority of trauma patients do not start on opioid-based medications until they suffer trauma, and other than preoperative use and fracture type there are no other demographic risk factors that surgeons should be screening for. Using the risk factors for opioid dependence identified through our study, future studies could develop risk calculators to help guide surgeons to optimize pain management postoperatively while mitigating risks of dependence.

While this study provides some important insights into the factors that influence postoperative opioid dependence after PHFs, it is not without limitations. Specifically, our opioid use data was collected from state-reported database and prescription data, the premise of our conclusions was based on the assumption that patients took their medications as prescribed. Although this may not always be the case, this is the most accurate method available to track opioid usage. In addition, while fractures were classified based on morphology, no examination of other injuries was included in this analysis which could have influenced opioid usage. In addition, we do not have any correlation to how opioid consumption affected outcomes following surgery. Finally, we only included surgically treated proximal humerus fractures, and the results cannot be extrapolated to non-operative or patients treated with an arthroplasty for their PHF. However, our goal was to focus our study with a specific PHF population to allow the most clear risk analysis of this population that could be utilized in future risk screening tools for surgical patients treated for a PHF.

## Conclusions

Despite these limitations, some important conclusions can be drawn from our results. Primarily, it is clear that preoperatively narcotic-dependent patients with complex fractures are at a higher risk postoperative dependence, and if opioids are used, there needs to be an appropriate plan to limit amounts and support a gradual weaning off of these medications in the immediate postoperative period. Preoperative risk assessments including prior opioid use, fracture complexity, and smoking status may help surgeons recognize patients at increased risk for postoperative opioid dependence and support appropriate postoperative weaning plans from opioids. Future studies should focus on alternate non-opioid-based treatment plans for pain management following surgical treatment of PHF as well as how opioid dependence influences patient satisfaction and outcomes.

## Data Availability

The datasets used and/or analyzed during the current study are available from the corresponding author on reasonable request.

## Abbreviations

ASA: American Society of Anesthesiologists
BMI: Body mass index
ORIF: Open reduction and internal fixation
PHF: Proximal humerus fractures
